# The Therapeutic Effects of SP-8356, a Verbenone Derivative, with Multimodal Cytoprotective Mechanisms in an Ischemic Stroke Rat Model

**DOI:** 10.3390/ijms252312769

**Published:** 2024-11-27

**Authors:** Hwa Young Song, Sejong Jin, Sekwang Lee, Angela Melinda Anthony Jalin, Kyung-Hye Roh, Won-Ki Kim

**Affiliations:** 1 Department of Neuroscience, Korea University College of Medicine, Seoul 02841, Republic of Koreaholicer@korea.ac.kr (S.J.); sklee@kumc.or.kr (S.L.);; 2 Central Research Institute, Shin Poong Pharmaceutical Company, Ltd., Ansan 15610, Republic of Korea; 3 Department of Anesthesiology and Pain Medicine, Korea University Ansan Hospital, Korea University College of Medicine, Ansan 15355, Republic of Korea; 4 Department of Physical Medicine and Rehabilitation, Korea University College of Medicine, Seoul 02841, Republic of Korea

**Keywords:** ischemic stroke, middle cerebral artery occlusion (MCAO), cytoprotective, SP-8356, verbenone, oxidative stress, inflammation, blood–brain barrier (BBB), brain edema

## Abstract

An ischemic cerebral stroke results from the interruption of blood flow to the brain, triggering rapid and complex cascades of excitotoxicity, oxidative stress, and inflammation. Current reperfusion therapies, including intravenous thrombolysis and mechanical thrombectomy, cause further brain injury due to reperfusion-induced cytotoxicity. To date, novel cytoprotective therapies that could address these challenges have yet to be developed, likely due to the limitations of targeting a single pathologic mechanism. To address these unmet clinical needs, we investigated a synthetic verbenone derivative, SP-8356, as a potential multi-target cytoprotective agent for acute ischemic strokes. In transient middle cerebral artery occlusion (MCAO) rats, SP-8356 significantly reduced brain infarct and edema volumes while improving acute neurological deficits in a dose-dependent manner. Furthermore, SP-8356 improved long-term outcomes, particularly by reducing mortality. These potent cytoprotective effects of SP-8356 were achieved by suppressing the excessive production of free radicals and pro-inflammatory cytokines, reducing the infiltration of inflammatory cells, and mitigating increases in blood–brain barrier permeability. Additional research is needed to determine whether co-administration of SP-8356 can extend the therapeutic time window of reperfusion therapies by mitigating ischemia/reperfusion injury.

## 1. Introduction

Ischemic stroke is one of the leading causes of death and long-term disability worldwide [1]. It occurs due to the interruption of blood flow to the brain, resulting in brain injury. Thus, restoring cerebral perfusion is a crucial therapeutic strategy to eliminate the cause of ischemic strokes. Currently, intravenous thrombolysis using recombinant tissue plasminogen activator (tPA) and mechanical thrombectomy for removing large artery thrombosis are the standard methods of reperfusion therapy [2]. However, reperfusion therapy can only salvage viable tissue in the peri-infarct region before irreversible damage occurs from ischemia. Moreover, reperfusion therapy carries the risk of further brain injury due to reperfusion injury, limiting the therapeutic time window and indications due to reperfusion-induced complications [3]. To address this challenge, additional cytoprotective drugs are needed to reduce ischemia/reperfusion injury [4].

The pathophysiology of ischemic strokes involves complex cascades of excitotoxicity, oxidative stress, and inflammation following ischemia/reperfusion [5]. Despite extensive research on cytoprotective drugs to reduce ischemia/reperfusion injury, no single mechanism targeting drug has achieved satisfactory clinical outcomes [6], likely due to this chaotic nature of ischemic strokes. Since multiple damaging mechanisms proceed simultaneously, inhibiting just one mechanism may not be sufficient [7]. This highlights the importance of developing multi-target-directed therapeutic approaches [8,9].

**Figure 1:**
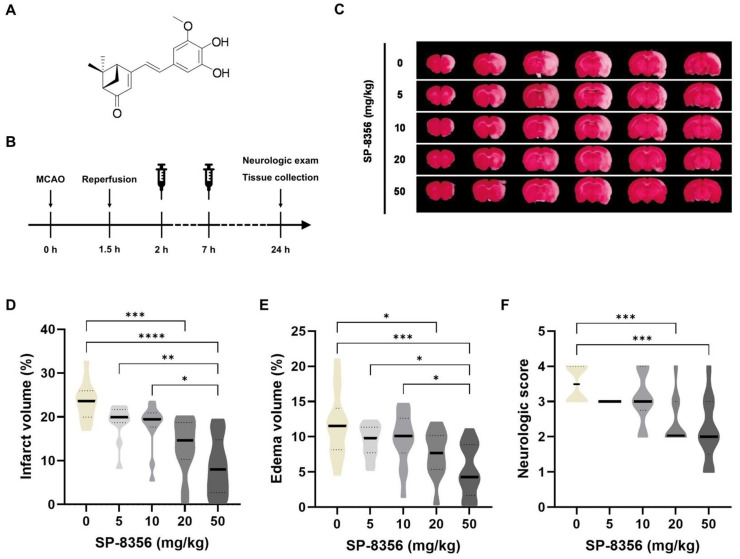
The cytoprotective effects of SP-8356 against acute ischemia/reperfusion brain injury. (**A**) The chemical structure of SP-8356. (**B**–**F**) SP-8356 (0 to 50 mg/kg, *n* = 12–14 per group) was administered intraperitoneally at 2 and 7 h post-MCAO, and outcomes were measured 24 h post-MCAO. (**B**) The flow chart. (**C**) Representative TTC-stained coronal sections of MCAO rat brains. (**D**) Infarct volume, (**E**) edema volume, and (**F**) neurologic score are presented as violin plots, with solid and dashed lines indicating median and interquartile range (Q1 to Q3, IQR), respectively. An amount of 0 mg/kg of SP-8356 is the indicated vehicle administration. Data were analyzed using one-way ANOVA followed by Tukey’s post hoc test or the Kruskal–Wallis test followed by Dunn’s test. * *p* < 0.05, ** *p* < 0.01, *** *p* < 0.001, **** *p* < 0.0001 compared with the indicated groups. MCAO, middle cerebral artery occlusion; TTC, 2,3,5-Triphenyltetrazolium chloride.

Previously, we developed (1S,5R)-4-(3,4-dihydroxy-5-methoxystyryl)-6,6-dimethylbicyclo [3.1.1] hept-3-en-2-one (SP-8356, Figure 1A) by adding potent free radical scavenging activity and NMDA receptor antagonist properties [10] to verbenone, a well-known natural anti-inflammatory compound [11,12]. As expected, SP-8356 showed superior cytoprotective efficacy in an in vitro experimental stroke model (oxygen/glucose deprivation) [10]. In addition, SP-8356 has demonstrated therapeutic efficacy in various disease models. SP-8356 showed anti-atherosclerosis effects by inhibiting the cluster of differentiation 147 (CD147)/matrix metalloproteinase 9 (MMP9) pathway [13,14] and anti-metastatic effects on breast and liver cancers by inhibiting the NF-κB signaling pathway [15,16]. SP-8356 also showed therapeutic efficacy in acute inflammatory lung injury induced by LPS [17] and in alkali-burned rat corneas [18]. These pharmacological effects were found to be closely related to the anti-inflammatory and cell migration inhibitory effects of SP-8356. Recently, a phase 1 clinical trial has been conducted to evaluate its clinical applicability in atherosclerosis. Considering that the inflammatory response and MMP activation are implicated in ischemic strokes, these results suggest that SP-8356 hold potential for stroke treatment. However, its efficacy under in vivo ischemic conditions has not yet been validated. Therefore, this study aimed to evaluate the anti-ischemic effects of SP-8356 in a rat model of transient middle cerebral artery occlusion (MCAO).

## 2. Results

### 2.1. SP-8356 Shows Cytoprotective Effects Against Acute Ischemia/Reperfusion Brain Injury

We first investigated the therapeutic effects of SP-8356 against ischemia/reperfusion injury, assessed 24 h after MCAO (Figure 1B–F). Overall, SP-8356 demonstrated dose-dependent cytoprotective effects, reducing infarct volume (Figure 1D), edema volume (Figure 1E), and neurologic deficit (Figure 1F). Notably, the 50 mg/kg dose of SP-8356 showed the most favorable outcomes. Considering the importance of both cortical and deep brain structures in neurological outcomes [19], we further analyzed the efficacy of SP-8356 in the cortex and subcortex separately. The results indicated that SP-8356 treatment significantly reduced infarct volume in both regions (Supplementary Figure S1). Additionally, the administration of 50 mg/kg did not result in significant change in the physiological parameters (Supplementary Table S1). Based on these results, a dose of 50 mg/kg was selected for the subsequent experiments.

### 2.2. SP-8356 Improves Long-Term Outcomes

**Figure 2:**
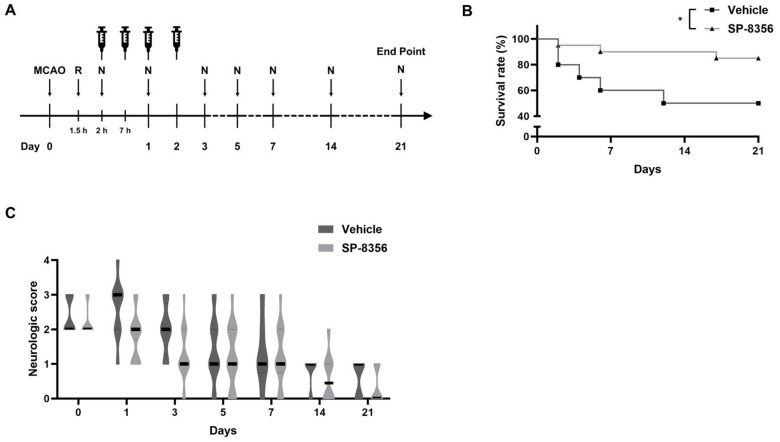
The long-term outcomes of SP-8356 treatment in MCAO rats. Vehicle (*n* = 10) or SP-8356 (50 mg/kg, *n* = 20) were administered intraperitoneally at 2, 7, 24, and 48 h post-MCAO, and outcomes were monitored for 21 days. (**A**) The flow chart. (**B**) The daily survival rates are presented as Kaplan–Meier curves and analyzed using the log-rank test. (**C**) The neurologic scores of surviving MCAO rats on 0, 1, 3, 5, 7, 14, and 21 days are presented as violin plots, with solid and dashed lines indicating median and IQR, respectively. Day 0 refers to 2 h post-MCAO. Data were analyzed using Mann–Whitney U test, followed by Bonferroni correction for adjusting multiple comparison at each time point. No statistically significant differences were observed after correction. * *p* < 0.05 compared with the indicated groups. MCAO, middle cerebral artery occlusion; R, reperfusion; N, neurologic examination.

To evaluate the long-term outcomes of SP-8356 treatment, we monitored survival time and neurologic scores in MCAO rats over a period of 21 days after MCAO. SP-8356 treatment improved survival outcomes compared to the vehicle treatment (Figure 2B), demonstrating better survival rates and longer mean survival times. On the final day (21 days post-MCAO), there were no significant differences in neurologic scores (Figure 2C) among the surviving animals. When neurologic scores were analyzed across all animals (including the deceased animals, which were scored as 7 [20]), the SP-8356 group showed lower scores compared to the vehicle group (vehicle: 4 (1–7) vs. SP-8356: 0 (0–1), uncorrected *p* = 0.035). However, this difference did not reach statistical significance after Bonferroni correction for multiple comparisons. Overall, SP-8356 treatment appears to improve long-term outcomes, particularly in terms of survival rate, but no statistically significant effects were observed in alleviating neurologic deficits.

### 2.3. SP-8356 Alleviates Oxidative/Nitrosative Stress in Ischemia/Reperfusion Injury

**Figure 3:**
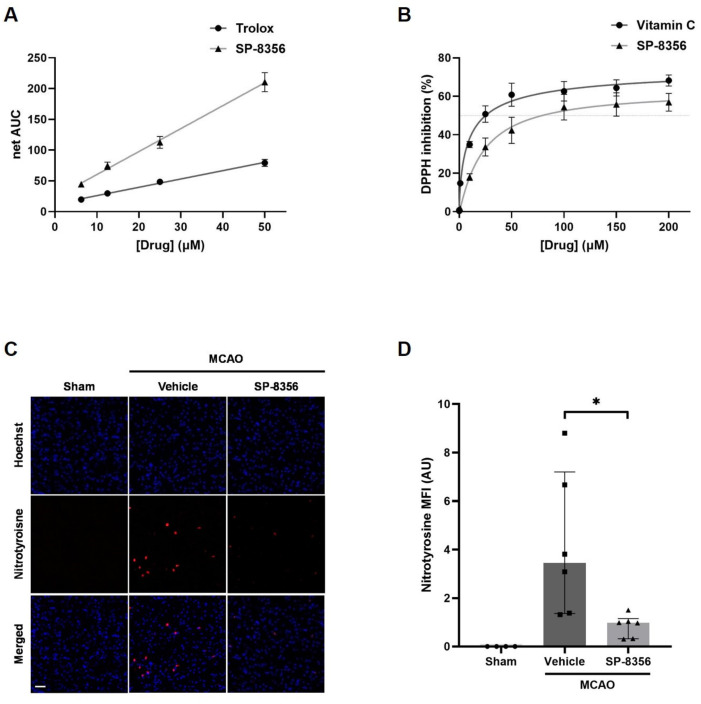
The anti-oxidative/nitrosative capacity of SP-8356 in vitro and in vivo. (**A**) The ORAC assay (*n* = 6). Data are presented as mean ± SEM, and linear fit curves according to concentration of SP-8356 and Trolox are shown. (**B**) The DPPH assay (*n* = 3). Dose–response curves of SP-8356 or vitamin C are shown. Data are presented as mean ± SEM. (**C**,**D**) The immunofluorescence staining of nitrotyrosine (red) and Hoechst (blue) in the peri-infarct area of the ischemic brain hemisphere (*n* = 4–6). (**C**) Representative images of nitrotyrosine immunofluorescence. Scale bar = 50 µm. (**D**) The quantification of nitrotyrosine in the ipsilateral hemisphere. Data are presented as median ± IQR. A statistical analysis was performed using the Kruskal–Wallis test followed by Mann–Whitney U test with Bonferroni correction. * *p* < 0.05 compared with the indicated groups. MCAO, middle cerebral artery occlusion; AUC, area under curve; MFI, mean fluorescence intensity; AU, arbitrary units.

Since oxidative stress is one of the major mechanisms of ischemic injury, we investigated the antioxidative efficacy of SP-8356. The free radical scavenging capacity of SP-8356 was assessed using an oxygen radical absorbance capacity (ORAC) assay and a 1,1-diphenyl-2-picrylhydrazyl (DPPH) assay. The results showed that SP-8356 scavenged peroxyl radicals in a dose-dependent manner, being 2.66 times more effective than Trolox at 50 μM (Figure 3A). Additionally, SP-8356 inhibited the release of organic nitrogen radicals in a dose-dependent manner, with its capacity at 50 µM being about 70% that of vitamin C, another well-known antioxidant (Figure 3B).

Next, we examined the in vivo effects of SP-8356 on oxidative stress in MCAO rats by comparing the immunoreactivity of nitrotyrosine, a well-known marker of oxidative/nitrosative stress in ischemic animal models [21]. The results showed reduced levels of nitrotyrosine in the SP-8356-treated group (Figure 3C,D). Overall, SP-8356 demonstrated antioxidant capacity in brains damaged by ischemia/reperfusion.

### 2.4. SP-8356 Reduces Inflammatory Responses in the Ischemic Lesions

**Figure 4:**
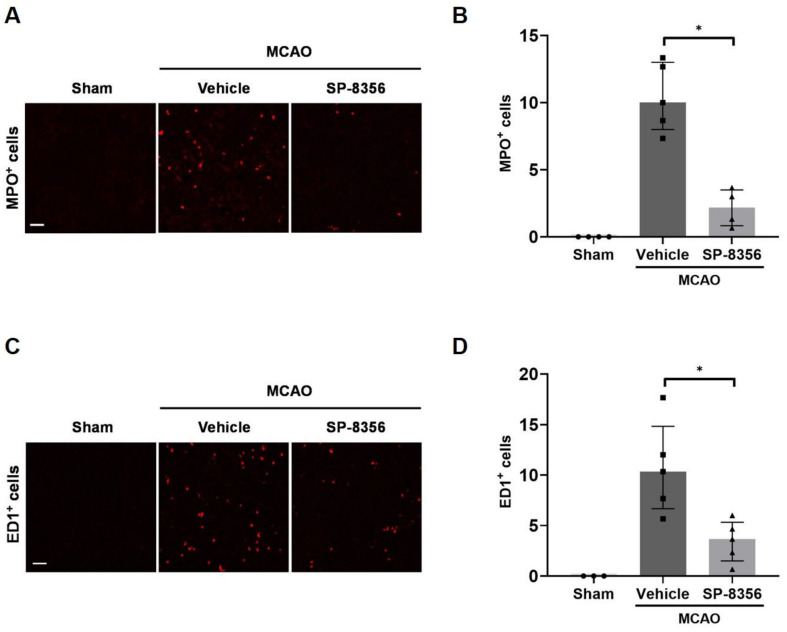
Inhibitory effects of SP-8356 on the infiltration of inflammatory cells into the ipsilateral brain lesion. Immunofluorescence staining in the peri-infarct area of the ischemic brain hemisphere of MPO (**A**,**B**, *n* = 4–5) and ED1 (**C**,**D**, *n* = 3–5). (**A**,**C**) Representative images of MPO-positive (**A**) or ED1-positive (**C**) cells. Scale bar = 50 µm. (**B**,**D**) The quantification of MPO-positive (**B**) or ED1-positive (**D**) cell counts in the ipsilateral hemisphere. Data are expressed as median ± IQR. Statistical comparison was conducted using the Kruskal–Wallis test followed by the Mann–Whitney U test with Bonferroni correction. * *p* < 0.05 compared with the indicated groups. MCAO, middle cerebral artery occlusion.

Excessive inflammatory responses after ischemia are known to play a crucial role in pathogenesis and prognosis [22]. Recruited peripheral leukocytes into the ischemic brain are known to aggravate neuroinflammation and tissue damage [23]. Therefore, we first investigated the infiltration of neutrophils and monocytes, which are the initial responders to the brain ischemia [24]. The number of MPO- or ED1-positive cells (specific markers for neutrophils or monocytes/macrophages, respectively) increased significantly in the ischemic regions due to MCAO, indicating that peripheral circulating immune cells infiltrate the ischemic lesion. Moreover, SP-8356-treated rats showed a lower number of MPO-positive cells compared with vehicle-treated rats (Figure 4B) and ED1-positive cells (Figure 4D).

**Figure 5:**
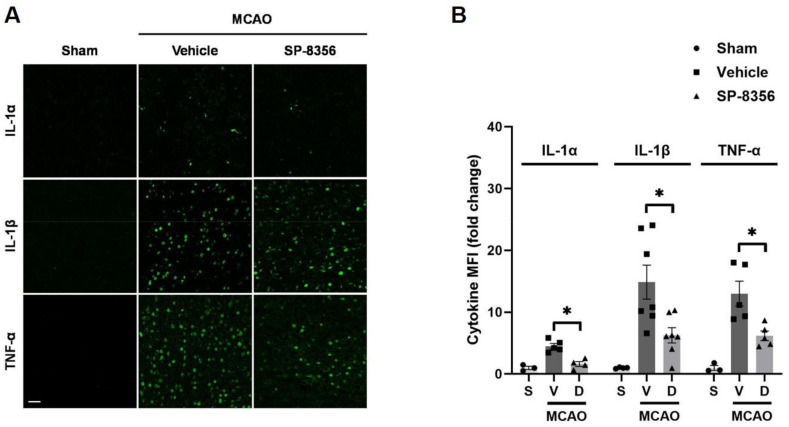
Inhibitory effects of SP-8356 on pro-inflammatory cytokines in the ipsilateral brain lesion. Immunofluorescence staining in the peri-infarct area of the ischemic brain hemisphere of IL-1α (*n* = 3–5)-, IL-1β (*n* = 4–7)-, and TNF-α (*n* = 3–5)-producing cells. (**A**) Representative images of IL-1α-, IL-1β-, and TNF-α-producing cells. Scale bar = 50 µm. (**B**) The quantification of cytokine MFI in the ipsilateral hemisphere. Data are shown as median ± IQR and were statistically analyzed using the Kruskal–Wallis test followed by the Mann–Whitney U test with Bonferroni correction. * *p* < 0.05 compared with the indicated groups. MCAO, middle cerebral artery occlusion; MFI, mean fluorescence intensity; S, sham-operated group; V, vehicle-treated group; D, SP-8356-treated group.

Additionally, the overexpression of pro-inflammatory cytokines can exacerbate damage in ischemic strokes [25]. SP-8356 treatment reduced the overexpression of pro-inflammatory cytokines induced by MCAO such as IL-1α, IL-1β, and TNF-α (Figure 5).

### 2.5. SP-8356 Prevents BBB Disruption Induced by Ischemia/Reperfusion Injury

**Figure 6:**
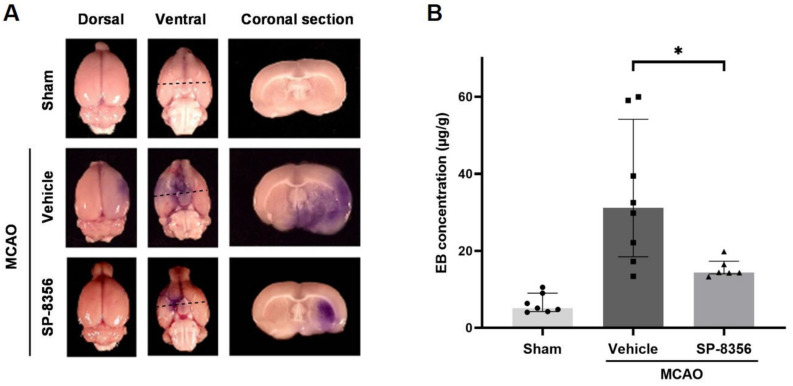
BBB-protective effects of SP-8356 in the ischemic brain region. EB extravasation in brains 24 h post-MCAO (*n* = 6–8). (**A**) Representative images of brain images after EB administration. The blue stained area means increased permeability of the BBB. The dashed line in the ventral image indicates the location of the coronal section. (**B**) The quantification of EB concentration in the ipsilateral hemisphere. Data are presented as median ± IQR. A statistical analysis was performed using Welch’s ANOVA followed by Games–Howell post hoc test. * *p* < 0.05 compared with the indicated groups. MCAO, middle cerebral artery occlusion; EB, Evans blue dye; BBB, blood–brain barrier.

Damage to the blood–brain barrier (BBB) induced by ischemia/reperfusion injury causes increased edema, hemorrhage, and the infiltration of inflammatory cells [26]. Therefore, we investigated BBB permeability by measuring the extravasation of intravenously injected Evans blue (EB) in the injured lesion. The results showed that the SP-8356 treatment significantly reduced EB leakage in the ipsilateral hemisphere, indicating SP-8356 provides BBB protection (Figure 6).

## 3. Discussion

Our study demonstrates that SP-8356 significantly improves both acute and long-term outcomes in a transient cerebral ischemic stroke model. SP-8356 reduced both cortical and subcortical infarctions, decreased brain edema, alleviated neurological deficits, and improved long-term survival, possibly through its antioxidant, anti-inflammatory, and BBB protection properties. This multi-target-directed anti-ischemic efficacy of SP-8356 suggests its potential as a therapeutic agent for ischemic strokes.

SP-8356 was designed to have superior antioxidant capacity by incorporating modified styryl, methoxy, and hydroxyl moieties, which are well known for their antioxidant properties [10,27,28]. Given the temporal profile of the pathophysiological cascade following acute focal cerebral ischemia [29], addressing early oxidative stress should be a critical step in preventing the onset of ischemic damage. In this study, SP-8356 demonstrated antioxidant activity both in vitro and in vivo.

Various antioxidants have been explored and shown promising therapeutic efficacy in preclinical studies [30]. However, clinical trials have demonstrated that simple treatment with antioxidants alone may not be sufficient to provide cytoprotective effects [6]. For example, uric acid, an antioxidant, showed no overall benefits in the general population [31], although it exhibited therapeutic effects in specific subgroups [32,33,34]. This suggests that multi-target agents may hold more promise, as therapeutic responses to single-target agents can be heterogeneous.

In our study, SP-8356 effectively reduced the infiltration of inflammatory cells. However, it remains unclear whether this effect is due to the direct inhibition of inflammatory cell migration or is secondary to reduced brain injury. Our previous studies have shown that SP-8356 inhibits leukocyte adhesion and migration [13,14,17,35]. SP-8356 acts as an inhibitor of CD147/MMP9 pathway [13,14], which is known to play a crucial role in cell migration [36]. Based on these studies, it is likely that SP-8356 inhibits leukocyte infiltration directly. The inhibitory effect of SP-8356 on pro-inflammatory cytokines may also be explained by its previously observed effect on the NF-kB pathway in response to LPS-induced inflammation [17], specifically by inhibiting the nuclear translocation of p65 [15,16].

The infiltration of circulating inflammatory cells into brain tissue during ischemic injury is intrinsically linked to the permeability of the blood–brain barrier (BBB). In the present study, SP-8356 inhibited the increase in BBB permeability caused by ischemic injury. Increased BBB permeability typically leads to post-ischemic cerebral edema, leukocyte infiltration, and secondary hemorrhagic transformation [37]. Our study observed significant reductions in edema volume (Figure 1E) and inhibition in leukocyte infiltration (Figure 4) in ischemic lesion. The mechanism by which SP-8356 reduces BBB leakage is not yet fully understood. However, the degradation of tight junction proteins by MMPs is a well-known mechanism of BBB disruption in ischemic strokes [38,39]. Our previous studies have shown that SP-8356 suppresses MMP9 activity under various conditions [13,14,15,16,18] by regulating CD147 dimerization [13] and CD147-cyclophilin A interaction [14], both of which are upstream of MMP expression. Therefore, MMP9 inhibition by SP-8356 may contribute to its BBB-protective effects, although this requires further clarification in future studies.

The anti-ischemic efficacy of SP-8356 observed in this study indicates multi-target direct ligand properties beyond a single-target mechanism. Currently, several multi-target cytoprotective drugs like SP-8356 are being developed for the treatment of ischemic strokes. First, minocycline, a tetracycline-class antibiotic, has been reported to have anti-ischemic properties through its antioxidant, anti-inflammatory, and anti-excitotoxic functions [40]. Meta-analyses indicate that minocycline shows beneficial effects in both preclinical and clinical studies [41,42]. Nelonemdaz (Neu2000) is an NMDA receptor antagonist and antioxidant [43]. A phase 2 study confirmed its safety in patients undergoing endovascular thrombectomy within 12 h [44], and a phase 3 study is currently underway [45]. Otaplimastat (SP-8203) is a cytoprotective agent with antioxidant [46], anti-excitotoxic [47], and BBB protective effects [48]. A phase 2 study of otaplimastat has been recently completed in patients who received intravenous thrombolysis within 4.5 h of stroke onset, and a phase 3 study is planned.

SP-8356 showed anti-ischemic properties through multi-targeted mechanisms, showing positive outcomes in the acute phase and reduced 21-day mortality in a MCAO rat model. However, in long-term neurologic sequelae, the SP-8356-treated group showed no significant difference compared to the vehicle-treated group. The higher mortality in the vehicle-treated group may have led to the dropout of subjects with severe brain damage, which may have blunted the observable effects of SP-8356 on long-term neurologic recovery. Additionally, subtle long-term functional improvements may not be detected due to the insensitivity of the simple neurological scoring system used. Symptoms assessed by this neurological scoring system are known to resolve rapidly during the early post-infarction phase [49], which is consistent with the trend observed in our study. Future studies should consider using more sensitive assessments to better evaluate long-term outcomes.

In this experiment, SP-8356 was dissolved by adding dimethyl sulfoxide (DMSO) and Cremophor EL^®^ to saline because of its low solubility in water. We are currently exploring various prodrugs by modifying SP-8356 to increase its solubility in water for clinical application.

In conclusion, our findings suggest that SP-8356 is a promising therapeutic candidate for ischemic strokes. Its multi-targeted mechanisms, including antioxidant, anti-inflammatory, and BBB protective effects, offer significant potential for improving outcomes in acute ischemic strokes, thereby addressing a critical unmet clinical need. Future work will be needed to demonstrate the therapeutic efficacy of combination therapy with SP-8356 and tPA in an embolic stroke model using blood clots.

## 4. Materials and Methods

### 4.1. Reagents and Antibodies

SP-8356 was synthesized by ShinPoong Pharmaceutical Co., Ltd. (Ansan, Republic of Korea). 2,3,5-triphenyltetrazolium chloride (TTC) was purchased from Alfa Aesar (Haverhill, MA, USA). 6-hydroxy-2,5,7,8-tetramethylchroman-2-carboxylic acid (Trolox), vitamin C, Hoechst 33258, 1,1-diphenyl-2-picrylhydrazyl (DPPH), dimethyl sulfoxide (DMSO), Cremophor EL^®^, and all other chemicals were obtained from Sigma-Aldrich (St. Louis, MO, USA). The following antibodies were used: anti-nitrotyrosine antibody from Millipore (Temecula, CA, USA); anti-ED1 and anti-Il-1α antibodies from Serotec (Oxford, UK); anti-myeloperoxidase (MPO) antibody from Dako Cytomation (Glostrup, Denmark); and anti-IL-1β and anti-TNF-α antibodies from Abbiotec (San Diego, CA, USA). Alexa 593 goat anti-mouse and Alexa 488 goat anti-rabbit secondary antibodies were obtained from Molecular Probes (Eugene, OR, USA).

### 4.2. Animals

Adult male Sprague–Dawley rats (260–300 g) were obtained from Charles River Laboratories International (Wilmington, MA, USA) and maintained on a 12 h light/dark cycle with unrestricted access to food and water. The rats were acclimated to the housing environment for one week prior to experiments. All procedures followed the National Institutes of Health (NIH) guidelines for the care and use of laboratory animals (7th Edition) and received approval from the Institutional Animal Care and Use Committee (IACUC) at Korea University College of Medicine (Approval No: KOREA-2016-0069).

During all surgical procedures, rats were initially anesthetized via facemask with 3% isoflurane in an N_2_O and O_2_ (7:3, *v*/*v*) mixture and then maintained with 2% isoflurane. Body temperature was maintained at 37 ± 0.5 °C on a heating pad and monitored with a rectal thermometer throughout the surgical period.

### 4.3. Transient Intraluminal Middle Cerebral Artery Occlusion (MCAO) Rat Model

Focal cerebral ischemia was induced by right-side MCAO using an intraluminal filament, as previously described with slight modification [50]. This method involves blocking the origin of the MCA with a filament to induce ischemia in the MCA territory, followed by filament removal to allow for reperfusion, thus mimicking ischemia/reperfusion injury of ischemic stroke. It is one of commonly used animal models for studying strokes [51].

Briefly, a heat-blunted 4-0 nylon monofilament was inserted into the lumen of the right external carotid artery (ECA) and advanced 19 mm into the internal carotid artery (ICA) from the bifurcation between the ECA and ICA to achieve MCAO. After 90 min of occlusion, the filament was removed to allow for reperfusion. Sham-operated animals were subjected to the same procedure but without the monofilament insertion.

Two hours after MCAO, only rats that showed signs of stroke (i.e., flexion of the contralateral forelimb with or without contralateral circling) were included in the studies and were randomly assigned to each experimental group. Rats that showed any abnormal signs, including seizures after the induction of stroke, were excluded from the study.

### 4.4. Drug Administration

SP-8356 (5 to 50 mg/kg) was dissolved in 5% DMSO, 10% Cremophor EL^®^, and 85% sterile saline (*v*/*v*/*v*). The drugs were intraperitoneally injected twice at 2 and 7 h after the onset of MCAO. In the long-term study, either vehicle or SP-8356 (50 mg/kg) was administered intraperitoneally at 2, 7, 24, and 48 h after MCAO. The drug dosage was determined based on our pilot study.

### 4.5. Physiological Monitoring

Left femoral artery catheterization was performed for intra-arterial blood pressure monitoring and blood collection. Physiological variables were measured 15 min before MCAO and 60 min after the first drug administration. Mean arterial blood pressure (MABP) was measured using a DigiMed blood pressure analyzer (Micro-Med, Louisville, KY). Blood pH, arterial partial O_2_ pressure (PaO_2_), and arterial partial CO_2_ pressure (PaCO_2_) were measured using iSTAT^®^ automated pH/blood gas analyzer with CG8^+^ cartridges (iSTAT, Abbott Lab., Abbott Park, IL, USA).

### 4.6. Measurement of Brain Infarct and Edema Volume

Brain infarct volume in rat MCAO model was measured as described below. Briefly, 24 h after MCAO, the brains were extracted following anesthesia and decapitation. Extracted brains were cut into 2 mm coronal sections using a rat brain matrix (Ted Pella, Redding, CA, USA) and stained with 2% TTC in phosphate-buffered saline (PBS) for 15 min to visualize the infarcted area. The brain infarct area was determined using a digital image analysis program, TOMORO Scope Eye v3.5 (Techsan Digital Co., Seoul, Republic of Korea). The cortex and subcortex were anatomically identified using the rat brain atlas [52]. Brain edema was assessed by the following calculation: % edema = [(V_I_ − V_C_)/V_C_] × 100, where V_I_ and V_C_ are the ipsilateral (affected) and the contralateral (unaffected) hemisphere volumes, respectively. The total volume of brain infarction was corrected for edema, as described previously [53]. Corrected infarct volume (IV_C_, mm^3^) was calculated using the following equation: IV_C_ = IV_d_ × (V_C_/V_I_), where IV_d_ is the ipsilateral infarct volume by direct measurement. The percentage of infarct volume was calculated using the following equation: infarct volume (%) = IV_C_/V_W_ × 100, where V_W_ is the whole brain volume.

### 4.7. Assessment of Neurologic Deficits

Neurologic deficits were evaluated by scoring on an 7-point scale neurologic score as previously described [20]. Neurologic scores were determined based on the following criteria: “0 = no neurological deficit; 1 = failure to extend right forepaw fully; 2 = decreased grip of the right forelimb while tail gently pulled; 3 = spontaneous movement in all directions, contralateral circling only if pulled by the tail; 4 = circling or walking to the right; 5 = walks only when stimulated; 6 = unresponsive to stimulation with a depressed level of consciousness; and 7 = dead” [20]. Neurologic deficits were scored 24 h after MCAO. In the long-term study, neurological evaluations were conducted at 1, 3, 5, 7, 14, and 21 days after MCAO.

### 4.8. Immunofluorescence

Twenty-four hours after MCAO, the brain was extracted from anesthetized rats, fixed with 4% paraformaldehyde (PFA), and then embedded in optimal cutting temperature (OCT) compound on dry ice after cryoprotection with phosphate buffer containing 30% sucrose. OCT-embedded brains were cut into 8–12 µm coronal sections using a cryostat microtome (Leica, CM1850, Wetzlar, Germany). The sections were washed with PBS and then incubated in blocking buffer (5% normal goat serum in PBS) for 1 h at room temperature. Sections were incubated overnight at 4 °C with following primary antibodies: anti-ED-1 (1:100), anti-MPO (1:800), anti-IL-1α (1:200), anti-IL-1β (1:100), anti-TNF-α (1:200), and anti-nitrotyrosine (1:150) in blocking buffer. Subsequently, the brain sections were washed with PBS to remove remaining primary antibodies and incubated with Alexa-594 or Alexa-488 fluorochrome-conjugated secondary antibodies for 2 h at room temperature. After counterstaining with Hoechst 33258 (1:1000), sections were mounted with fluorescence mounting medium (DAKO, Glostrup, Denmark). The fluorescent immunoreaction of the antigen was visualized with a confocal laser scanning microscope (Zeiss, LSM 510, Oberkochen, Germany). Quantitative analysis was performed on three non-overlapping regions of interest (ROIs) in the peri-infarct area of the ischemic brain hemisphere. The number of marker-positive cells and mean fluorescence intensity (MFI) was quantified using ImageJ software (National Institutes of Health, Bethesda, MD, USA). The MFI for each cytokine was standardized to the mean value of the sham group and presented accordingly.

### 4.9. Evans Blue (EB) Leakage

To evaluate blood–brain barrier (BBB) permeability, a 2% EB (Sigma-Aldrich, St. Louis, MO, USA) solution in 0.1 M PBS was injected intravenously into the tail vein at the onset of reperfusion according to a previously described method [54] and then allowed to circulate for 24 h. The brains were isolated after transcardiac perfusion with sterile saline to remove all intravascular blood and homogenized in 50% trichloroacetic acid. EB was extracted by incubating the homogenized samples overnight at 4 °C, followed by centrifugation at 10,000× *g* for 15 min. The amount of EB dye in the supernatant was measured at 610 nm using a spectrophotometer (SpecraMAX90, Molecular Device, Sunnyvale, CA, USA) and expressed as µg/g per ipsilateral hemisphere.

### 4.10. Oxygen Radical Absorbance Capacity (ORAC) Assay

The ORAC assay, an analytical method to evaluate free radical scavenging capacity based on the reaction of antioxidants against peroxyl radicals, was performed as previously described [55]. Briefly, 50 nM fluorescein were pre-incubated at 37 °C for 10 min, then a peroxyl radical generator, 2,2′-azobis-(2-methylpropionamide)-dihydrochloride (AAPH, 60 mM), was added. The decrease in fluorescence intensity was measured every 5 min for 9 h at 37 °C using a fluorescence microplate spectrophotometer (Ex = 485 nm, Em = 530 nm; SpectraMax GeminiEM, Molecular Devices, Sunnyvale, CA, USA). For the quantification of antioxidant effects against peroxyl radicals, the area under the curve (AUC) of each sample was calculated using the following equation: $AUC=\left(\;0.5+\sum_{i=1}^{n}\frac{f_{i}}{f_{0}}\;\right)\times 5$, where *f_i_* is the fluorescence intensity at the *i*-th time point. The net AUC_sample_ was determined by subtracting the AUC_blank_ from the AUC_sample_: $net{AUC}_{sample}={AUC}_{sample}-{AUC}_{blank}$.

### 4.11. 1,1-Diphenyl-2-picrylhydrazyl (DPPH) Assay

The free radical scavenging effect of SP-8356 was measured using the DPPH assay as previously described with slight modification [56]. In brief, drug samples were mixed with an organic nitrogen radical generator, DPPH solution (23.6 μg/mL in ethanol), at 37 °C for 30 min in the dark. After 3 h of incubation to reach a stable state, the absorbance (Abs) of the remaining DPPH was measured at 517 nm using a microplate spectrophotometer (SpectraMax 340 PC; Molecular Devices, Sunnyvale, CA, USA). Radical scavenging capacity was calculated as DPPH inhibition (%): $DPPHinhibition\left(\%\right)=\left({Abs}_{control}-{Abs}_{sample}\right)/{(Abs}_{control})\times 100$.

### 4.12. Statistical Analysis

Data are expressed as mean ± standard error of the mean (SEM) or median with interquartile range (Q1 to Q3, IQR). Normality was assessed using the Shapiro–Wilk test, and homogeneity of variances was tested using the Brown–Forsythe test. For comparisons among groups, one-way ANOVA followed by Tukey’s post hoc test, Welch’s ANOVA followed by Games–Howell test, or Kruskal–Wallis test followed by Dunn’s test or Mann–Whitney U test was performed. Multiple comparisons were adjusted using the Bonferroni correction. Survival analysis was performed using the Kaplan–Meier method with the log-rank test. A *p*-value of less than 0.05 was considered statistically significant. All analyses were conducted using an IBM SPSS Statistics 26 (IBM, Armonk, NY, USA) and GraphPad Prism 10 (GraphPad Software, San Diego, CA, USA).

## Data Availability

The data presented in this study are available on request from the corresponding author.

## Supplementary Materials

The following supporting information can be downloaded at https://www.mdpi.com/article/10.3390/ijms252312769/s1.
