# Supplementary material for: The Therapeutic Effects of SP-8356, a Verbenone Derivative, with Multimodal Cytoprotective Mechanisms in an Ischemic Stroke Rat Model
Source: Int J Mol Sci. 2024 Nov 27;25(23):12769. doi: 10.3390/ijms252312769 (PMC11641512; doi:10.3390/ijms252312769)
Supplement: Supplementary file 1 [file ijms-25-12769-s001.zip › ijms-3285099-supplementary.pdf]

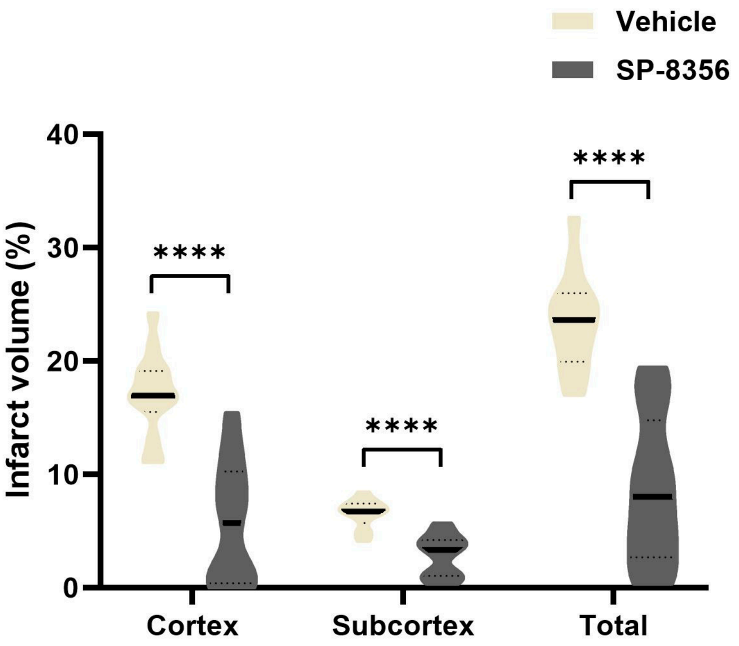

Supplementary Figure S1. The cytoprotective effect of SP-8356 on the cortex and subcortex regions. The region-specific analysis of infarct volume in the vehicle (n = 14) and SP-8356 (50 mg/kg, n = 13) groups shown in Figure 1. The cortex and subcortex region were anatomically identified using the rat brain atlas. Data are presented as violin plots, with solid and dashed lines indicating median and interquartile range (Q1 to Q3, IQR). A statistical analysis was performed using the Mann–Whitney U test. **** $p < 0.0001$ compared with the indicated groups.

Supplementary Table S1. Physiological variables.

	Temp (°C)	pH	PaO_2 (mmHg)	$PaCO_2$ (mmHg)	MABP (mmHg)
			15 minutes before MCAO		
**Vehicle**	36.85 ± 0.38	7.41 ± 0.06	165.80 ± 7.66	47.28 ± 9.31	104.45 ± 7.26
**SP-8356 (50 mg/kg)**	36.88 ± 0.34	7.44 ± 0.03	167.60 ± 6.69	43.86 ± 3.68	103.75 ± 4.48
			60 minutes after 1st drug treatment		
**Vehicle**	37.95 ± 0.37	7.39 ± 0.10	158.20 ± 24.53	44.64 ± 7.26	96.35 ± 8.54
**SP-8356 (50 mg/kg)**	37.52 ± 0.80	7.43 ± 0.04	172.20 ± 11.03	40.70 ± 7.38	100.85 ± 8.92

Physiological variables were measured 15 minutes before MCAO and 60 minutes after 1st treatment with vehicle or SP-8356. Temp, rectal temperature; PaO_2, partial arterial pressure of oxygen; $PaCO_2$, partial arterial pressure of CO_2; MABP, mean arterial blood pressure. Values are expressed as means ± SD (n = 5 per group).
